# Ocean acidification increases the accumulation of titanium dioxide nanoparticles (nTiO_2_) in edible bivalve mollusks and poses a potential threat to seafood safety

**DOI:** 10.1038/s41598-019-40047-1

**Published:** 2019-03-05

**Authors:** Wei Shi, Yu Han, Cheng Guo, Wenhao Su, Xinguo Zhao, Shanjie Zha, Yichen Wang, Guangxu Liu

**Affiliations:** College of Animal Sciences, Zhejiang University, Hangzhou, P.R. China

## Abstract

Large amounts of anthropogenic CO_2_ in the atmosphere are taken up by the ocean, which leads to ‘ocean acidification’ (OA). In addition, the increasing application of nanoparticles inevitably leads to their increased release into the aquatic environment. However, the impact of OA on the bioaccumulation of nanoparticles in marine organisms still remains unknown. This study investigated the effects of OA on the bioaccumulation of a model nanoparticle, titanium dioxide nanoparticles (nTiO_2_), in three edible bivalves. All species tested accumulated significantly greater amount of nTiO_2_ in *p*CO_2_-acidified seawater. Furthermore, the potential health threats of realistic nTiO_2_ quantities accumulated in bivalves under future OA scenarios were evaluated with a mouse assay, which revealed evident organ edema and alterations in hematologic indices and blood chemistry values under future OA scenario (pH at 7.4). Overall, this study suggests that OA would enhance the accumulation of nTiO_2_ in edible bivalves and may therefore increase the health risk for seafood consumers.

## Introduction

Over the past century, increased CO_2_ emissions derived from anthropogenic activities have led to measurable declines in both oceanic pH and the abundance of carbonate ions, a process commonly termed ‘ocean acidification’^1^. The average global surface ocean pH has significantly decreased by over 0.1 units since the Industrial Revolution and is expected to further decline by 0.3 units by the end of the century^2,3^. This projected increase in seawater *p*CO_2_ has been identified to exert far-reaching impacts on marine ecosystems and negatively affect a wide range of physiological processes across a large diversity of marine organisms^2,4–10^.

Nanotechnology is a rapidly growing field attracting considerable attention owing to its widespread applications in both consumer and industrial products^11,12^. It has been predicted that the global market for nanotechnology products would achieve $3 trillion by 2020^12^. Consequently, this rapid expansion and widespread application would inevitably be accompanied by a heightened release of nanoparticles into the environment, posing potential threats to both environment and human health. For example, the US Environmental Protection Agency (USEPA) has attributed 60,000 deaths per year to the inhalation of atmospheric nanoparticles^13^. Due to precipitation and surface runoff, marine ecosystems are regarded as the ultimate sink for nanoparticles^14,15^. To date, much research has shown that nanoparticle contamination can exert significant impacts on the biological and physiological processes of various marine organisms, including fertilization^16^, protein expression^17^, immune responses^18^, energy budgets^19^ and even survival^20^. In addition, previous studies have illustrated that nanoparticles can bio-accumulate in marine invertebrate species and are bio-amplified through the food chain^21–24^. Therefore, consuming nanoparticle-contaminated seafood could be a potential risk for human health. So far, the health consequences of exposure to nanoparticle contaminations have been well studied in mammals^13^. For example, evident liver lesions, kidney pathological alterations^25^ and hampered immune responses^26^ induced by oral titanium dioxide nanoparticles (nTiO_2_) exposure have been described in mice. Though toxicity studies of nanoparticles have been conducted *in vivo* in mouse models, excessive doses application in these previous studies have failed to precisely reflect the health consequences of consuming nanoparticle-contaminated food^27^. Therefore, the current understanding of nanoparticle-induced food safety issues remains limiting.

It has been demonstrated that water pH can affect surface charging properties and hence the aggregation, potential bioavailability and reactivity of nanoparticles^28–31^. Therefore, theoretical future ocean acidification scenarios may alter the uptake and bioavailability of nanoparticles in marine systems and subsequently determine the critical degree of nanoparticle contaminations. A recent study has indicated that elevated atmospheric CO_2_ levels would modify the effects of nTiO_2_ on the nutritional quality of crops with unknown consequences for human health^32^; whether oceanic *p*CO_2_ increase would aggravate or attenuate the risk of consuming seafood harvested in nanoparticle polluted areas awaits investigation.

Compared with other nanoparticles (Cu, Ag, Fe, ZnO nanoparticles, etc.), nTiO_2_ has the highest model-predicted concentrations in the environment, which may reach as high as several mg/L in the aquatic system^33,34^. Therefore, nTiO_2_ has been widely used as a representative nanoparticle in ecotoxicological studies^35–37^. With little known about food safety risk induced by nanoparticle contamination, specifically under future ocean acidification scenarios and at environmentally realistic concentrations, the present study was conducted using nTiO_2_ as a model nanoparticle with three edible bivalve species to address the following questions: (1) Does elevated *p*CO_2_ alter the ingestion-related physicochemical properties of nanoparticle in seawater? (2) How will nanoparticle accumulation in bivalves be affected by future ocean acidification scenarios? and (3) How and to what extent elevated *p*CO_2_ in seawater will affect human’s risk via consuming seafood contaminated with environmentally realistic nanoparticle concentrations.

## Results

### Effects of ocean acidification on the ingestion-related physicochemical properties of nTiO_2_

Compared to the particle sizes (334.8 nm in diameter) in seawater at an ambient pH of 8.1, larger sizes of nTiO_2_ (439.8 and 537.1 nm in diameter at pH of 7.8 and 7.4, respectively) were detected in *p*CO_2_ acidified seawater. The zeta potentials of nTiO_2_ (the charge of nTiO_2_ in relation to the surrounding conditions) increased with decreasing seawater pH, being most stable in seawater at pH 8.1 due to the large negative zeta potential (Table 1). Since it has been reported that nanoparticles in larger aggregates are easier to be ingested^38^, the particle size alteration detected, which may partially result from zeta potential change, implies increased likelihood of nTiO_2_ to be ingested by bivalves under ocean acidification scenarios.

**Table 1:** Physicochemical properties of titanium dioxide nanoparticles (mean ± SE).

Property	nTiO_2_
Average diameter	30 ± 5 nm
BET surface area	60.65 m^2^ g^−1^
Crystal structure	
Purity	99.8%
Hydrodynamic diameter*	
in pH 8.1 seawater	334.8 ± 6.7 nm^a^
in pH 7.8 seawater	439.8 ± 11.2 nm^b^
in pH 7.4 seawater	537.1 ± 13.6 nm^c^
Zeta potential*	
in pH 8.1 seawater	−10.7 ± 0.1 mV^a^
in pH 7.8 seawater	−9.8 ± 0.1 mV^b^
in pH 7.4 seawater	4.9 ± 0.1 mV^c^

Mean values that do not share the same superscript were significantly different at *p* < 0.05.

^*^The particle hydrodynamic diameters and zeta potential were tested at a dose of 0.1 mg L^−1^ nTiO_2_.

### Effects of ocean acidification on the accumulation of nTiO_2_ in various tissues of three edible bivalve species

**Figure 1:**
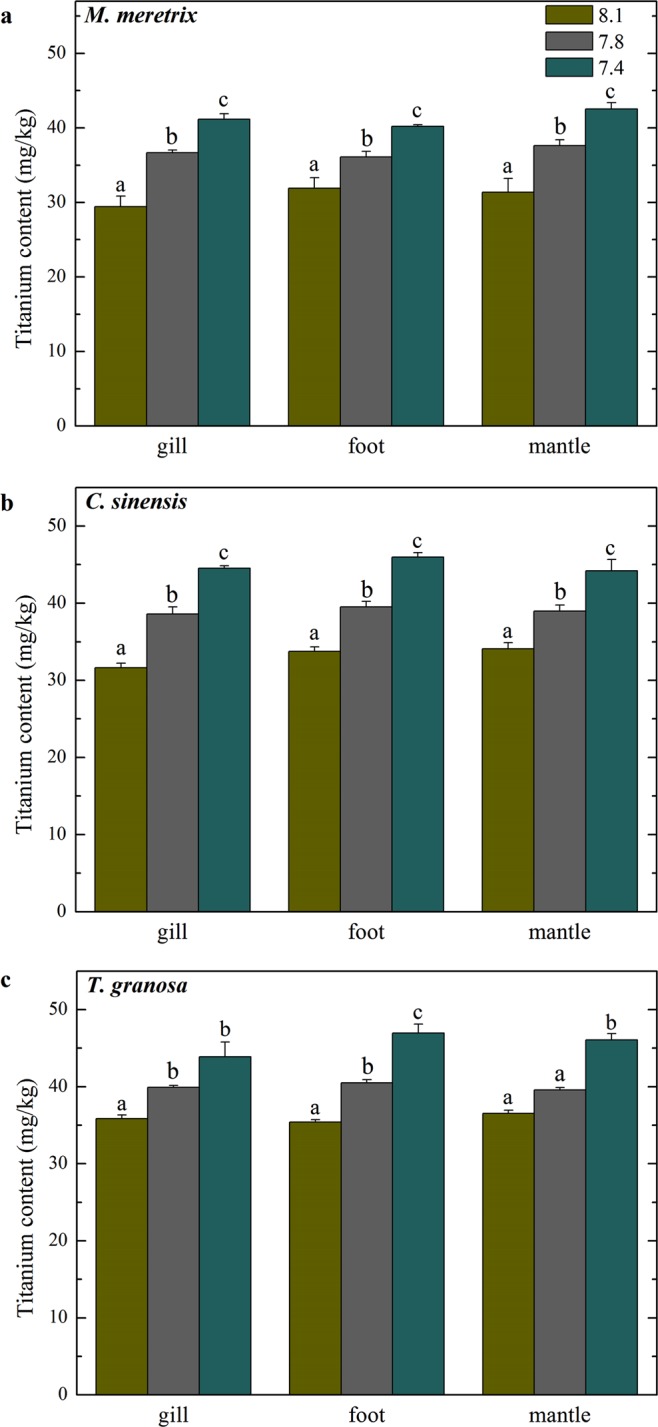
TiO_2_ concentration (mean ± SE) (mg/kg, dry wet) in different tissues of (**a**) *M. meretrix*, (**b**) *C. sinensis*, and (**c**) *T. granosa* reared in different *p*CO_2_ levels (21 days exposure). Mean values that do not share the same superscript were significantly different at *p* < 0.05.

The nTiO_2_ contents accumulated in the gills, foot and mantles of blood clam (*Tegillarca granosa*), hard clam (*Meretrix meretrix*), and venus clam (*Cyclina sinensis*) after a 21-day 100 μg/L nTiO_2_ exposure at pH 7.4 and 7.8 were about 1.34 and 1.16 times greater than those raised in the ambient pH of 8.1, respectively (Fig. 1, *p* < 0.05). This finding suggests that ocean acidification increases the accumulation of nTiO_2_ in the bivalve species (Table S1).

### The impacts of ocean acidification on seafood safety: mouse assays

**Figure 2:**
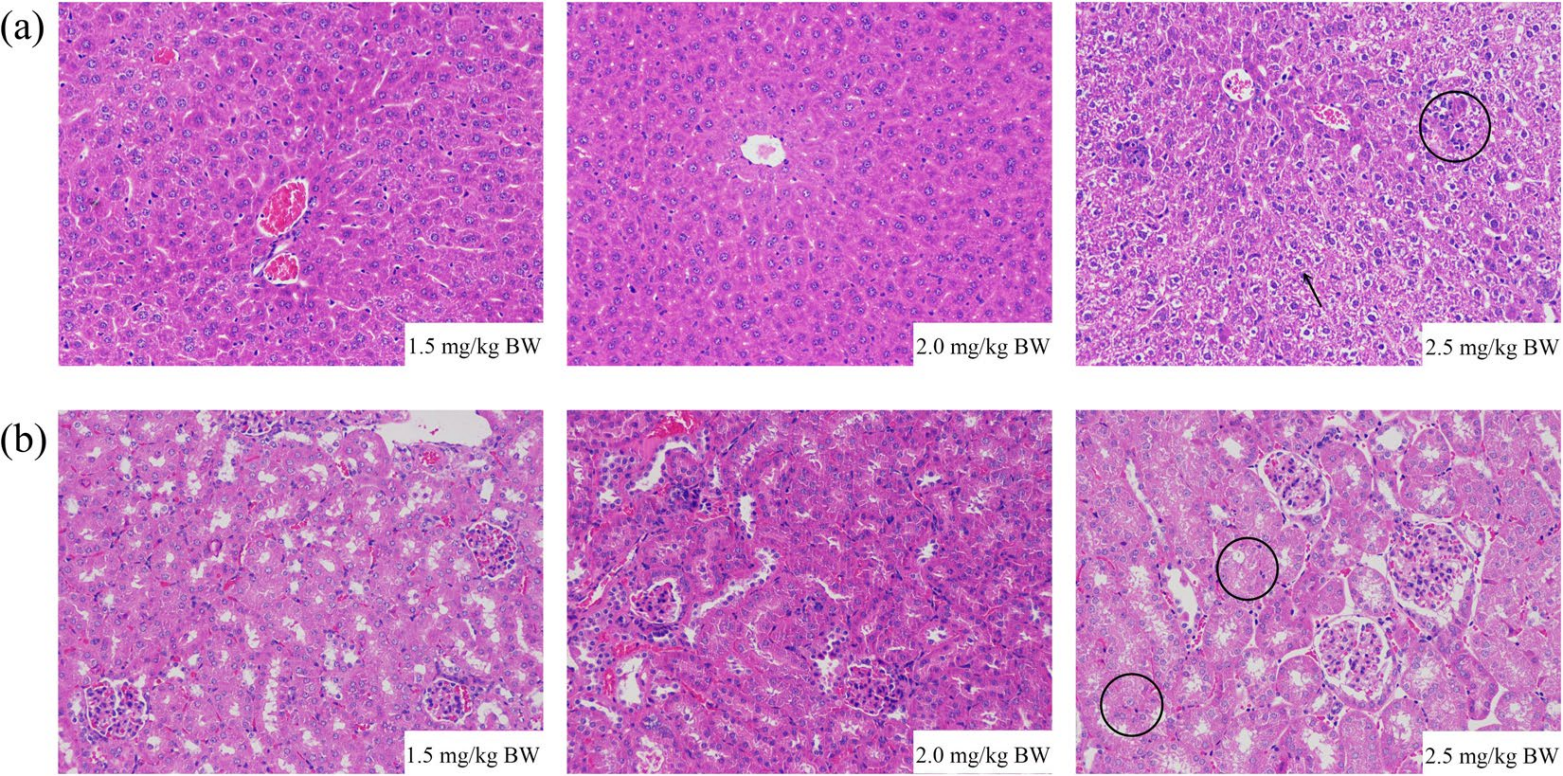
Representative histological photomicrographs of liver (**b**) and kidney (**b**) in mice after exposure to nTiO_2_ at different doses corresponding to daily intake of nTiO_2_-contaminated seafood at different *p*CO_2_ levels for 30 days (HE × 200). Three mice in each group were used for histological examination (n = 3). (**a**) Inflammatory cell infiltration (circles) and edema (arrows) were observed in the 2.5 mg/kg BW group. (**b**) Renal tubular epithelial cells were slightly swollen (circles) in the 2.5 mg/kg BW group. No obvious pathological changes were found in the heart, spleen and lung among the different treatment groups.

Upon feeding with 2.5 mg/kg nTiO_2_ per day, an equivalent dose simulating the intake of nTiO_2_ through consuming contaminated bivalves raised under pH 7.4, the numbers of white blood cell (WBC) and lymphocytes (Lym) in mice were significantly greater than those under ambient pH of 8.1 (Table 2). Similarly, the levels of alanine transaminase (ALT), alkaline phosphatase (ALP), and creatinine (Crea), as well as the ALT/aspartate transaminase (AST) ratio were significantly induced upon feeding with an equivalent amount of nTiO_2_ accumulated in the bivalves raised under ocean acidification scenarios (Table 3). Evident liver edema indicated by sinusoidal expansion (arrows in Fig. 2a) and inflammatory cell infiltration (or hydropic degeneration) (circles in Fig. 2a) was detected in mice fed with 2.5 mg/kg nTiO_2_ daily (Fig. 2a). In addition, inflammatory cell infiltration and slight swelling of renal tubular epithelial cells (circles in Fig. 2b) were also observed in the kidneys of these mice.

**Table 2:** Hematologic indices of mice after oral exposure to nTiO_2_ at different exposure doses corresponding to daily intake of nTiO_2_-contaminated seafood at different *p*CO_2_ levels for 30 days (mean ± SE).

Group	Exposure dose	WBC (10^9^/L)	Lym (10^9^/L)	Mon (10^9^/L)	Gran (10^9^/L)	Mon %	RBC (10^12^/L)	MCH (pg)
pH 8.1	1.5 mg/kg BW	9.9 ± 1.3^a^	7.0 ± 0.7^a^	0.3 ± 0.1^a^	2.6 ± 0.6^a^	3.4 ± 0.4^a^	8.6 ± 0.9^a^	16.5 ± 0.4^a^
pH 7.8	2 mg/kg BW	10.3 ± 0.6^a^	6.9 ± 0.2^b^	0.4 ± 0.1^a^	2.9 ± 0.4^a^	3.8 ± 0.4^a^	8.7 ± 0.9^a^	17.2 ± 0.2^a^
pH 7.4	2.5 mg/kg BW	15.9 ± 1.2^b^	12.5 ± 1.1^c^	0.4 ± 0.0^a^	3.1 ± 0.2^a^	2.6 ± 0.2^a^	9.7 ± 0.6^a^	16.9 ± 0.4^a^
**Group**	**Exposure dose**	**RDW %**	**Gran %**	**Lym %**	**Hgb (g/L)**	**HCT %**	**MCV (fl)**	**MCHC (g/L)**
pH 8.1	1.5 mg/kg BW	12.6 ± 0.4^a^	25.6 ± 2.7^a^	71.0 ± 2.9^a^	142.2 ± 14.5^a^	42.8 ± 4.2^a^	50.1 ± 1.2^a^	330.6 ± 2.5^a^
pH 7.8	2 mg/kg BW	12.3 ± 0.4^a^	28.3 ± 2.5^a^	67.8 ± 2.7^a^	151.2 ± 16.3^a^	45.9 ± 5.0^a^	52.5 ± 0.7^a^	328.8 ± 1.4^a^
pH 7.4	2.5 mg/kg BW	12.3 ± 0.8^a^	19.3 ± 1.6^a^	78.1 ± 1.5^a^	163.2 ± 7.4^a^	49.8 ± 3.1^a^	51.5 ± 0.9^a^	329.7 ± 5.4^a^

WBC, white blood cell; Lym, lymphocytes; Mon, monocytes; Gran, granulocytes; RBC, red blood cells; MCH, mean corpuscular hemoglobin; RDW, red cell distribution width; Hgb, hemoglobin; MCV, Mean corpuscular volume; MCHC, mean corpuscular hemoglobin concentration. Mean values that do not share the same superscript were significantly different at *p* < 0.05.

**Table 3:** Blood chemistry values of mice after oral exposure to nTiO_2_ at different exposure doses corresponding to daily intake of nTiO_2_-contaminated seafood at different *p*CO_2_ levels for 30 days (mean ± SE).

Group	Exposure dose	ALT	AST	ALT/AST	ALP	Crea	Bun
pH 8.1	1.5 mg/kg BW	28.9 ± 1.4^a^	93.7 ± 7.4^a^	0.31 ± 0.01^a^	77.9 ± 5.5^a^	33.6 ± 0.3^a^	17.2 ± 0.9^a^
pH 7.8	2 mg/kg BW	34.0 ± 2.3^a^	103.5 ± 3.9^a^	0.33 ± 0.01^a^	110.0 ± 4.8^b^	34.2 ± 0.4^a^	18.0 ± 0.6^a^
pH 7.4	2.5 mg/kg BW	42.0 ± 3.1^b^	106.5 ± 3.2^a^	0.39 ± 0.02^b^	108.9 ± 6.5^b^	35.3 ± 0.4^b^	18.1 ± 0.5^a^

ALT, alanine transaminase; AST, aspartate transaminase; ALP, alkaline phosphatase; BUN, blood urea nitrogen; Crea, creatinine. Mean values that do not share the same superscript were significantly different at *p* < 0.05.

The body weights of mice increased continuously throughout the experiment (Fig. S1), and no significant differences were detected among groups fed with different amounts of nTiO_2_ (equivalent intake doses through consuming contaminated bivalves raised under ambient and two ocean acidification scenarios, respectively) (Table S2). The coefficients of heart, liver, spleen, lung, and kidney with body weight did not significantly differ among groups (Table S2). No obvious pathological changes were detected in the heart, spleen and lung of mice exposed to various tested doses of nTiO_2_ (Fig. S2).

## Discussion

Seafood, including edible bivalves, contributes almost 20% of animal proteins for over three billion people in the world^39^. However, the presence of contaminants in seafood poses threats to consumers’ health and therefore draws increasing attention from both the scientific community and regulatory authorities, such as the Food and Agriculture Organization (FAO)^39^, the Food and Drug Administration (FDA)^40^ and the European Food Safety Authority (EFSA)^41^. The fast growth of the production and application of nanoparticles in a variety of products will inevitably add onto the release of nanoparticles into the environment, which can accumulate in marine organisms, including commercial seafood species, posing potential threats to human health^22,24,42^. Although some evidence suggests that seafood safety may worsen under future climate scenarios^7,43^, the plausible effects of future ocean acidification scenarios on the safety of seafood with respect to nanoparticle contaminations have been largely overlooked.

The present study showed that future ocean acidification scenarios will lead to an increased accumulation of nTiO_2_ in marine edible bivalves, which may be due to a synergic effect of *p*CO_2_-driven ocean acidification on the uptake of nTiO_2_ from the environment and the exclusion of nTiO_2_ out of the body of bivalves. First, ocean acidification may induce nTiO_2_ accumulation by changing the aggregate size of nTiO_2_ in seawater. In aqueous solution, nanoparticles tend to aggregate forming large particles and the extent of aggregation is dependent on various factors including the surface charge of nanoparticle and the pH value of medium^29–31^. It has been shown that marine bivalves capture and ingest larger nanoparticle aggregates more efficiently than those in suspension^38,44^. For example, particles larger than 4 μm in diameter can be easily ingested by bivalve species through the process of filter feeding. However, the nanoparticle uptake efficiency of bivalves through this filtration process decreases asymptotically with the decreasing particle size of the nanoparticle^45^. According to previous studies, the pH at the point of zero charge (pH_zpc_) of nanoparticles has important implications for their aggregation, as electrostatic repulsion between nanoparticles of similar potential decreases when the solution pH approaches the pH_zpc_^29,31,35^. Our data showed that the zeta potential of nTiO_2_ approach the critical pH_zpc_ value of 0 in *p*CO_2_-acidified seawater, which subsequently reduced the electrostatic repulsion between nTiO_2_ favoring the formation of larger aggregates. Therefore, enhanced nTiO_2_ accumulation under future ocean acidification scenarios could be partially due to easier ingestion of bigger aggregates by marine bivalves.

Second, the increase of nTiO_2_ accumulation in bivalves could also be attributed to an increase in nTiO_2_ uptake through other pathways. Theoretically, all substances in the marine environment could diffuse directly into the body via damaged tissues^46^. As increased *p*CO_2_ can bring about severe tissue damage to marine organisms^47,48^, ocean acidification may facilitate the entry of nanoparticles into marine organisms by causing tissue lesion. In addition, nanoparticles can also enter the extrapallial fluid between the soft tissues and shell of marine bivalves through incorporation into the nacreous layer^49^. As mantle and labial palps have been confirmed as the target organs for the internalization of nanoparticles in marine invertebrates^50^, nanoparticles deposited on the shell surfaces may be transported into the extrapallial fluid and subsequently accumulate in the body. Since ocean acidification can result in shell dissolution and nanoparticle deposition acceleration^5,51^, increases in shell permeability and nanoparticle deposition on the shell surface induced by *p*CO_2_ acidification would facilitate the uptake of nTiO_2_ through the shell-extrapallial fluid pathway.

Third, since some of the nanoparticles ingested will be excreted from the body^52^, a hampered nTiO_2_ exclusion induced by ocean acidification may also account for the increased nTiO_2_ accumulation. The exclusion of most exogenous substances is an energy-consuming process, and it has been shown that ocean acidification may constrain energy availability for toxicant metabolism in marine invertebrates^7,53^. Therefore, the exclusion of nanoparticle could be reduced to some extent in elevated *p*CO_2_ conditions, leading to an increase in nanoparticle accumulation.

Previous investigations on the impacts of nanoparticles on food safety, including nTiO_2_, were generally conducted with excessive doses^36^. In addition, although it has been suggested that nanoparticles can accumulate in marine organisms such as gastropods^54^, amphipods^20^, bivalves^42^ and fish^24^, the health consequences of oral exposure to nanoparticle-contaminated seafood at environmentally realistic concentrations remains unknown. Our data showed that a 30-day oral exposure to nTiO_2_ at the equivalent doses of seafood consumption under future ocean acidification scenarios can threaten health in terms of organ lesion and alterations in hematologic indices and blood chemistry values. For instance, increased WBC and lymphocyte counts indicated an inflammatory reaction upon feeding with nTiO_2_ at the dose equivalent to oral intake via consuming contaminated seafood under future ocean acidification scenarios^55^. In addition, evident injuries were detected in liver since the organ is the main target organ for nanoparticles via oral exposure^56–59^. Similar kidney and hepatic injuries in mice after gastrointestinal nTiO_2_ exposure has been observed previously, and it was suggested that these injuries are associated with alterations in inflammatory cytokine expression and reduction in detoxification of nTiO_2_^60^.

According to our results obtained, ocean acidification would induce the bioaccumulation of nTiO_2_ in marine edible bivalves and cause liver and kidney injuries in mice at the equivalent dose of seafood consumption, indicating an increased health risk by consuming seafood under future ocean acidification scenarios. We speculate that this health risk could be underestimated, given the relatively short accumulation duration (30 days) examined in the present study. In addition, both ocean acidification and nanoparticles have been reported to facilitate the accumulation of other toxic pollutants through complex interactions^9,61^, further increasing seafood safety risk. Though it is generally accepted that the concentrations of nanoparticles in the environment will grow steadily and reach as high as mg/L level in water system^33^, little is known about its environmental concentrations. Future studies should consider long-term effects of nanoparticles and pH in the context of future ocean acidification as well as populations in different geographic locations.

## Methods

This study was carried out in three steps namely collection of bivalves from Yueqing Bay (Wenzhou, China) and acclimation in the Qingjiang Station of Zhejiang Mariculture Research Institute (Wenzhou, China) in July 2016, followed by a bioaccumulation experiment under different pH scenarios (pH 7.8 and 7.4, representing ocean acidification scenarios predicted by IPCC in 2100 and 2300, respectively) during August 2016, and mice toxicology assays performed in Zhejiang University (Hangzhou, China) during September 2016 to evaluate human’s risk of consuming seafood contaminated with nanoparticle.

### Collection and acclimation of bivalves

Adult *T. granosa* (29.6 ± 3.5 mm), *M. meretrix* (50.6 ± 4.7 mm) and *C. sinensis* (36.0 ± 4.4 mm) of similar shell length outside their breeding seasons were obtained from Yueqing Bay (Wenzhou, China). In order to determine the TiO_2_ concentration in these bivalve species from sampling sites, 10 individuals of each species were analyzed and TiO_2_ concentrations in all bivalve species were found to be under detection limits (0.3 ng/mg). To obtain the background concentration of TiO_2_ in the seawater, 10 mL of seawater samples was analyzed and TiO_2_ concentrations in these samples were 2.7 ± 0.4 μg/L. The TiO_2_ concentrations in both tissues and seawater samples were determined following the methods of the National Standard of China (Determination of titanium in foods, GB5009.246-2016)^62^. Prior to experiments, bivalves were acclimatized in sand-filtered seawater (pH 8.10 ± 0.03, salinity 21.3 ± 0.6‰) at an ambient water temperature 25.3 ± 0.5 °C with constant aeration and a natural light/dark cycle for at least 7 days. Bivalves were fed daily with microalgae (*Tetraselmis chuii*) at the satiation feed rate during the acclimation, and selected bivalves were cultured overnight in filtered seawater without feeding before the exposure experiment.

### *p*CO_2_-driven seawater acidification

Sand-filtered seawater from the clam-sampling site, Yueqing Bay, was used throughout the experiment. To simulate the present and near-future projections of the Intergovernmental Panel on Climate Change (IPCC), one ambient present pH (pH at 8.1) and two lower pH levels (pH 7.8 and 7.4, representing ocean acidification scenarios in 2100 and 2300, respectively) were used in the present study^2,3^. Seawater at different pH values was achieved and maintained by continuous aeration with CO_2_-air mixture with corresponding predicted *p*CO_2_, which was obtained by mixing CO_2_-free air and pure CO_2_ gas at known flow rates using flow controllers (Fig. S3)^7,9^. Throughout the experiment, pH calibrated with standard US National Bureau of Standards buffers (pH_NBS_), salinity, temperature, total alkalinity (TA) and carbonate system parameters including dissolved inorganic carbon (DIC), aragonite saturation state (Ωara) and calcite saturation state (Ωcal) were monitored daily. Seawater pH was measured with a Sartorius PB-10 pH meter (Sartorius, Germany) with an accuracy of ±0.01. Salinity was measured using a conductivity meter (Multi 3410 WTW, Germany) with an accuracy of ±0.5% measurements. Total alkalinity measurements were performed by potentiometric titration with an automatic titrator system (SMTitrino 702, Metrohm). Throughout the experiment, seawater pH_NBS_ was measured and adjusted by pH/ORP controllers (PC-2110, House, China; pH fluctuations were controlled within 0.01 units) to maintain the desired pH. The carbonate system parameters were calculated from the measured pH, salinity, temperature and TA values using the open-source program CO2SYS, as described previously^10^. The pH fluctuations were within 0.01 units over the 21 days exposure in all tanks. Seawater carbonate chemistry parameters in each tank measured and calculated daily for all treatments during the exposure were summarized in Table 4.

**Table 4:** Carbonate chemistry variables of seawater during the experiment (mean ± SE).

Target pH	T (°C)	Sal (‰)	pH_NBS_	TA (μmol/kg)	*p*CO_2_ (μatm)	DIC (μmol/kg)	Ωara	Ωcal
8.1	25.3 ± 0.4	21.3 ± 0.3	8.10 ± 0.03	2074 ± 6	581 ± 13	1912 ± 6	2.31 ± 0.03	3.65 ± 0.05
7.8	25.1 ± 0.3	21.7 ± 0.3	7.82 ± 0.03	2094 ± 11	1188 ± 25	2026 ± 4	1.27 ± 0.02	2.00 ± 0.04
7.4	25.2 ± 0.2	21.4 ± 0.3	7.41 ± 0.02	2073 ± 9	3140 ± 22	2153 ± 10	0.54 ± 0.01	0.85 ± 0.02

T: temperature; Sal: salinity; TA: total alkalinity; *p*CO_2_: CO_2_ partial pressure; DIC: dissolved inorganic carbon; Ωara: aragonite saturation state; and Ωcal: calcite saturation state.

### Characterization of nanoparticles

In this study, nTiO_2_ were purchased from Shanghai Klamar Reagent Co. Ltd, China, and the size and shape of the nTiO_2_ particles were determined using transmission electron microscopy (TEM, JEM-1230, JEOL, Tokyo, Japan). Crystal structure of the particles was identified using X-ray powder diffractometry (XRD, Rigaku D/MAX 2550/PC, Tokyo, Japan), and surface area was measured through Brunauer-Emmett-Teller (BET) adsorption measurements (Micromeritic TriStarII 3020, Micrometritics Instrument Corp., Norcross, GA). The majority of nTiO_2_ used in this study was anatase crystals with irregular shapes and a surface area of 60.65 m^2^/g (Fig. S4 and Table 1). To minimize weighing errors and ensure concentration accuracy, a stock solution of 1 g/L nTiO_2_ was prepared daily by dispersing the nTiO_2_ in ultrapure water followed by sonication for 15 mins^57,63,64^. Test solutions of nTiO_2_ were prepared immediately prior to use by diluting the stock solution with 0.1-μm membrane-filtered seawater (pH 8.10, salinity 21.3‰). Particle hydrodynamic diameter and zeta potential of nTiO_2_ in seawater at different pH values (ambient pH 8.1, pH 7.8 and 7.4) were tested with the Zetasizer Nano ZS90 (Malvern Instruments Ltd, Malvern, UK).

### Bioaccumulation experiment

Since nTiO_2_ is mostly introduced into the environment via sewage discharge and it remains about 100 μg/L nTiO_2_ in the waste water effluents after treatment^33,36^, seawater in polluted areas could be contaminated at equivalent magnitudes, especially when the steady increase of environmental nTiO_2_ is taken into account. Therefore, 100 μg/L was chosen to simulate the environmental concentration of nTiO_2_ in the polluted areas in this study. After one week of acclimation, bivalves (90 individuals for each species) were randomly assigned into plastic tanks, with a total seawater volume of 30 L containing approximately 100 μg/L nTiO_2_ and maintained under the desired pH levels. In total, 27 experimental tanks and 270 bivalves (10 individuals per tank × 3 replicate tanks × 3 pH levels × 3 species) were used in the present study. Bivalves were fed with *T. chuii* twice a day during the experiment and the seawater was changed daily with corresponding pre-acidified seawater containing the desired concentration of nTiO_2_. No mortality was observed throughout the experimental period.

Following that of Johnston^65^, Hooper^66^, and Gaiser^67^, in the present study an exposure time of 21 days was adopted to avoid the effect of stress syndrome. After 21-day exposure to 100 μg/L nTiO_2_ at different pH levels, five live individuals of each species were randomly taken from each tank and purged in sand-filtered seawater overnight. After rinsing with ultrapure water, the bivalve individuals were dissected on ice and the gill, mantle and foot muscle of each individual were peeled off and stored separately at −20 °C for TiO_2_ residue analysis. TiO_2_ concentrations in the various tissues were calculated and expressed in mg/kg dry weight for each individual. Similarly, the entire soft body of bivalves was peeled off to determine TiO_2_ concentration (expressed in mg/kg wet weight), which was later used to calculate the equivalent oral exposure dose of nTiO_2_ used in the mice toxicology assays.

### Content analysis of nTiO_2_

To obtain working concentration of nTiO_2_ in the seawater for each experimental group (pH at 8.1, 7.8, and 7.4, respectively), 1 mL of seawater samples (three replicates for each experimental group) were taken after seawater renewal. In addition, approximately 0.1–0.3 g of each tissue or the entire soft body were used to determine the amount of accumulated nTiO_2_. The contents of TiO_2_ in both tissue and seawater samples were determined following that of the National Standard of China (GB5009.246-2016)^62^. In brief, the water and tissue samples were digested in ultrapure nitric acid overnight. After adding 0.5 mL H_2_O_2_, mixtures were heated with an electric heating plate until samples were completely digested, and the remaining nitric acid was removed until colorless and clear solutions were achieved. The solutions were diluted to 3 mL with 2% nitric acid and used for titanium concentration measurement using inductively coupled plasma atomic emission spectrometry (ICP-MS, PE NexION 300X, USA). Indium (20 ng/ml) was taken as the internal standard and the detection limit of titanium was 0.074 ng/ml. The background and working concentrations of nTiO_2_ in seawater during the 21-day experiment are listed in Table 5.

**Table 5:** Background and working Ti concentration (μg/L) at different pH levels (mean ± SE).

	background	pH 8.1	pH 7.8	pH 7.4
Target concentration	0	100	100	100
Ti concentration	2.7 ± 0.4	99.5 ± 3.5	97.3 ± 1.7	98.3 ± 1.7

### Mice toxicology assays

Healthy Kunming (KM) male mice (8 weeks old) were purchased from the Experimental Animals Center of Zhejiang University and housed in plastic laboratory animal cages in room conditions (20 ± 2 °C, 60 ± 10% relative humidity, under a 12 h light/dark cycle) for a week. During the acclimation, a commercial pellet diet and deionized water were available *ad libitum*. After 7 days of acclimation, 15 adult mice were equally divided into 3 treatment groups (n = 5): the pH 8.1 exposed group, the pH 7.8 exposed group, and the pH 7.4 exposed group. All experiments were approved by the Animal Care Committee of Zhejiang University and all methods were performed in accordance with the Guidelines for the Care and Use of Animals for Research and Teaching at Zhejiang University.

The obtained mean values of the amount of nTiO_2_ accumulated in *M. meretrix*, the lowest of the three bivalve species, were used to calculate oral exposure doses. According to previous dietary surveys and guidelines^68,69^, 150 g/person, equivalent to approximately 3 g/body weight (BW)/day, was used as the amount of daily intake of seafood. Based on the nTiO_2_ concentrations detected in bivalve *M. meretrix* in the present study (5.05, 6.77, and 8.30 mg/kg at pH 8.1, 7.8 and 7.4, respectively), the daily intake of dietary nTiO_2_ through consuming nTiO_2_ contaminated seafood raised under pH 8.1, 7.8, and 7.4 was estimated to be 0.015, 0.020, and 0.025 mg/kg, respectively. Taking the interspecies extrapolation into consideration^70^, a 100-fold dose of the human exposure was used for mice assays (1.5, 2.0, and 2.5 mg/kg BW, respectively). After 15-min sonication in ultrapure water, nTiO_2_ suspensions were given to mice by gavage once a day for 30 consecutive days. During the 30-day experiment, body weight was recorded every 5 days and any symptom or mortality was observed and recorded daily. After 30 days of nTiO_2_ exposure, all mice were weighed and sacrificed after anesthetization. Blood samples were collected from the femoral artery in the groin area. Serum was obtained by centrifuging blood at 3000 rpm for 15 min. Tissues and organs such as heart, kidney, spleen, lung and liver were excised and weighed. Tissue samples for histopathologic examination were fixed in 10% neutral buffered formalin.

After weighing, the coefficients of various tissues to the body weight were calculated as the ratio of tissues (wet weight, mg) to body weight (g). Blood component parameters were determined with an auto hematology analyzer (BC-2800Vet, Shenzhen, China). Liver function was evaluated based on the serum levels of ALT, AST and ALP. Nephrotoxicity was determined by blood urea nitrogen (BUN) and Crea, which were determined using an automated biochemical analyzer (Hitachi 7170 A, Tokyo, Japan). All histopathological observations were performed according to standard laboratory procedures. Tissues were embedded in paraffin, sliced into 5-μm thicknesses and placed onto glass slides. After hematoxylin-eosin (HE) staining, the slides were examined and images were taken using an optical microscope (Nikon Eclipse Ci-L, Tokyo, Japan). The identity and analysis of the pathology slides were blind to the pathologist.

### Statistical analyses

Differences in hydrodynamic diameter and zeta potential of nTiO_2_ in seawater at different pH levels (pH 8.1, 7.8, and 7.4) were compared by one-way analyses of variance (One-way ANOVAs) followed by post-hoc Tukey tests using OriginPro 9.0.

The nTiO_2_ concentrations accumulated in individuals were assessed using a linear mixed effects model with treatment pH as a fixed factor and the treatment tank as a random factor. In total, nine linear mixed effects models were performed for each tissue and species investigated (3 species × 3 tissues) using ‘R’ statistical package lme4 (R Development Core Team, 2012).

Differences in hematologic indices and blood chemistry values of the mice after oral exposure to different nTiO_2_ doses were evaluated using one-way ANOVAs followed by post-hoc Tukey tests using OriginPro 9.0. Percentage data (e.g. percentages of monocytes, granulocytes and lymphocytes) were arcsine-square root transformed prior to analysis to meet the assumption of a normal distribution^71^.

For all analyses, Levene’s test and Shapiro-Wilk’s test were performed using OriginPro 9.0 to verify homogeneity of variance and normality, respectively. All data were presented as mean ± standard error (SE) and a *p*-value at *p* < 0.05 was taken as statistically significant.

## Supplementary information

Supporting information

## References

[1] Caldeira K, Wickett ME (2003). Oceanography: anthropogenic carbon and ocean pH. Nature.

[2] Orr JC (2005). Anthropogenic ocean acidification over the twenty-first century and its impact on calcifying organisms. Nature.

[3] Ellis RP, Urbina MA, Wilson RW (2017). Lessons from two high CO_2_ worlds - future oceans and intensive aquaculture. Global Change Biol..

[4] Hoegh-Guldberg O (2007). Coral reefs under rapidclimate change and ocean acidification. Science.

[5] Doney SC, Fabry VJ, Feely RA, Kleypas JA (2009). Ocean acidification: the other CO_2_ problem. Annu. Rev. Mar. Sci..

[6] Liu S (2016). Ocean acidification weakens the immune response of blood clam through hampering the NF-kappa beta and toll-like receptor pathways. Fish Shellfish Immunol..

[7] Shi W (2016). Ocean acidification increases cadmium accumulation in marine bivalves: a potential threat to food safety. Sci. Rep..

[8] Su W (2017). Benzo[a]pyrene exposure under future ocean acidification scenarios weakens the immune responses of blood clam. Tegillarca granosa. Fish Shellfish Immunol..

[9] Zhao X (2017). Ocean acidification decreases mussel byssal attachment strength and induces molecular byssal responses. Mar. Ecol. Prog. Ser..

[10] Zhao X (2017). Ocean acidification adversely influences metabolism, extracellular pH and calcification of an economically important marine bivalve. Tegillarca granosa. Mar. Environ. Res..

[11] Bouwmeester H (2009). Review of health safety aspects of nanotechnologies in food production. Regul. Toxicol. Pharm..

[12] Roco MC (2011). The long view of nanotechnology development: the national nanotechnology initiative at 10 years. J. Nanopart. Res..

[13] Oberdörster G, Oberdörster E, Oberdörster J (2005). Nanotoxicology: An emerging discipline evolving from studies of ultrafine particles. Environ. Health Pers..

[14] Nowack B, Bucheli TD (2007). Occurrence, behavior and effects of nanoparticles in the environment. Environ. Pollut..

[15] Canesi L, Corsi I (2016). Effects of nanomaterials on marine invertebrates. Sci. Total Environ..

[16] Nielsen HD, Berry LS, Stone V, Burridge TR, Fernandes TF (2009). Interactions between carbon black nanoparticles and the brown algae: Inhibition of fertilization and zygotic development. Nanotoxicology.

[17] Gomes T, Pereira CG, Cardoso C, Bebianno MJ (2013). Differential protein expression in mussels *Mytilus galloprovincialis* exposed to nano and ionic Ag. Aquat. Toxicol..

[18] Gagné F (2008). Ecotoxicity of CdTe quantum dots to freshwater mussels: Impacts on immune system, oxidative stress and genotoxicity. Aquat. Toxicol..

[19] Muller EB, Hanna SK, Lenihan HS, Miller R, Nisbet RM (2014). Impact of engineered zinc oxide nanoparticles on the energy budgets of *Mytilus galloprovincialis*. J. Sea Res..

[20] Hanna, S. K., Miller, R. J., Muller, E. B., Nisbet, R. M. & Lenihan, H. S. Impact of engineered zinc oxide nanoparticles on the individual performance of *Mytilus galloprovincialis*. *PLoS One* **8** (2013). 10.1371/journal.pone.0061800 PMC3629123 23613941

[21] Parks AN (2013). Bioaccumulation and toxicity of single-walled carbon nanotubes to benthic organisms at the base of the marine food chain. Environ. Toxicol. Chem..

[22] Conway JR, Hanna SK, Lenihan HS, Keller AA (2014). Effects and implications of trophic transfer and accumulation of CeO_2_ nanoparticles in a marine mussel. Environ. Sci. Technol..

[23] Wang J, Wang WX (2014). Low bioavailability of silver nanoparticles presents trophic toxicity to marine medaka (*Oryzias melastigma*). Environ. Sci. Technol..

[24] Wang Z, Yin L, Zhao J, Xing B (2016). Trophic transfer and accumulation of TiO_2_ nanoparticles from clamworm (*Perinereis aibuhitensis*) to juvenile turbot (*Scophthalmus maximus*) along a marine benthic food chain. Water Res..

[25] Wang J (2007). Acute toxicity and biodistribution of different sized titanium dioxide particles in mice after oral administration. Toxicol. Lett..

[26] Duan Y (2010). Toxicological characteristics of nanoparticulate anatase titanium dioxide in mice. Biomaterials.

[27] Shukla RK, Kumar A, Vallabani NV, Pandey AK, Dhawan A (2014). Titanium dioxide nanoparticle-induced oxidative stress triggers DNA damage and hepatic injury in mice. Nanomedicine.

[28] Ridley MK, Hackley VA, Machesky ML (2006). Characterization and surface-reactivity of nanocrystalline anatase in aqueous solutions. Langmuir.

[29] Dunphy Guzman KA, Finnegan MP, Banfield JF (2006). Influence of surface potential on aggregation and transport of titania nanoparticles. Environ. Sci. Technol..

[30] Domingos RF, Tufenkji N, Wilkinson KI (2009). Aggregation of titanium dioxide nanoparticles: role of a fulvic acid. Environ. Sci. Technol..

[31] French RA (2009). Influence of ionic strength, pH, and cation valence on aggregation kinetics of titanium dioxide nanoparticles. Environ. Sci. Technol..

[32] Du W (2017). Elevated CO_2_ levels modify TiO_2_ nanoparticle effects on rice and soil microbial communities. Sci. Total Environ..

[33] Gottschalk F, Sun T, Nowack B (2013). Environmental concentrations of engineered nanomaterials: review of modeling and analytical studies. Environ. Pollut..

[34] Sun T, Bornhöft NA, Hungerbuehler K, Nowack B (2016). Dynamic probabilistic modelling of environmental emissions of engineered nanomaterials. Environ. Sci. Technol..

[35] Sharma VK (2009). Aggregation and toxicity of titanium dioxide nanoparticles in aquatic environment - a review. J. Environ. Sci. Heal. A.

[36] Bourgeault A (2015). The challenge of studying TiO_2_ nanoparticle bioaccumulation at environmental concentrations: crucial use of a stable isotope tracer. Environ. Sci. Technol..

[37] Guan X (2018). Neurotoxic impact of acute TiO_2_ nanoparticle exposure on a benthic marine bivalve mollusk. Tegillarca granosa. Aquat. Toxicol..

[38] Hull MS (2011). Filter-feeding bivalves store and biodeposit colloidally stable gold nanoparticles. Environ. Sci. Technol..

[39] Food and Agriculture Organization. *The State of World Fisheries and Aquaculture 2016. Contributing to food security and nutrition for all*(FAO, 2016).

[40] Food and Drug Administration. Fish and Fishery Products Hazards and Controls Guidance, https://www.fda.gov/downloads/food/guidanceregulation/ucm251970.pdf/. Assessed: 7, 2018.

[41] European Food Safety Authority (2009). Scientific opinion of the panel on contaminants in the food chain on a request from the European Commission on marine biotoxins in shellfish - Saxitoxin group. EFSA J..

[42] Gomes T (2012). Accumulation and toxicity of copper oxide nanoparticles in the digestive gland of *Mytilus galloprovincialis*. Aquat. Toxicol..

[43] Williamwl C (2010). Large-scale redistribution of maximum fisheries catch potential in the global ocean under climate change. Global Change Biol..

[44] Ward JE, Kach DJ (2009). Marine aggregates facilitate ingestion of nanoparticles by suspension-feeding bivalves. Mar. Environ. Res..

[45] Riisgård H (1988). Efficiency of particle retention and filtration rate in 6 species of Northeast American Bivalves. Mar. Ecol. Prog..

[46] Handy RD, Owen R, Valsami-Jones E (2008). The ecotoxicology of nanoparticles and nanomaterials: current status, knowledge gaps, challenges, and future needs. Ecotoxicology.

[47] Bibby R, Widdicombe S, Parry H, Spicer J, Pipe R (2008). Effects of ocean acidification on the immune response of the blue mussel *Mytilus edulis*. Aquat. Biol..

[48] Frommel AY (2012). Severe tissue damage in Atlantic cod larvae under increasing ocean acidification. Nat. Clim. Change.

[49] Zuykov M, Pelletier E, Demers S (2011). Colloidal complexed silver and silver nanoparticles in extrapallial fluid of *Mytilus edulis*. Mar. Environ. Res..

[50] Ma S, Lin DH (2013). The biophysicochemical interactions at the interfaces between nanoparticles and aquatic organisms: adsorption and internalization. Environ. Sci. Proc. Impacts.

[51] Petosa AR, Jaisi DP, Quevedo IR, Elimelech M, Tufenkji N (2010). Aggregation and deposition of engineered nanomaterials in aquatic environments: role of physicochemical interactions. Environ. Sci. Technol..

[52] Rocha TL, Gomes T, Sousa VS, Mestre NC, Bebianno MJ (2015). Ecotoxicological impact of engineered nanomaterials in bivalve molluscs: An overview. Mar. Environ. Res..

[53] Roberts DA (2013). Ocean acidification increases the toxicity of contaminated sediments. Global Change Biol..

[54] Li HY, Turner A, Brown MT (2013). Accumulation of aqueous and nanoparticulate silver by the marine gastropod *Littorina littorea*. Water Air Soil Poll..

[55] Tang HQ (2016). The effect of ZnO nanoparticles on liver function in rats. Int. J. Nanomedicine.

[56] He LS, Yan XS, Wu DC (1991). Age-dependent variation of zinc-65 metabolism in LACA mice. Int. J. Radiat. Biol..

[57] Wang Y (2013). Susceptibility of young and adult rats to the oral toxicity of titanium dioxide nanoparticles. Small.

[58] Sang X (2015). Immunomodulatory effects in the spleen-injured mice following exposure to titanium dioxide nanoparticles. J. Biomed. Mater. Res. A.

[59] Sang X (2013). Toxicological mechanisms of nanosized titanium dioxide-induced spleen injury in mice after repeated peroral application. J. Agr. Food Chem..

[60] Gui S (2011). Molecular mechanism of kidney injury of mice caused by exposure to titanium dioxide nanoparticles. J. Hazard. Mater..

[61] Zhu X, Zhou J, Cai Z (2011). TiO_2_ nanoparticles in the marine environment: impact on the toxicity of tributyltin to abalone (*Haliotis diversicolor supertexta*) embryos. Environ. Sci. Technol..

[62] Standardization Administration of China (SAC). China National Standards Compilation, GB5009.246-2016 (Standards Press of China, 2016).

[63] Petković J (2011). DNA damage and alterations in expression of DNA damage responsive genes induced by TiO_2_ nanoparticles in human hepatoma HepG2 cells. Nanotoxicology.

[64] Tian SY, Zhang YD, Song CZ, Zhu XS, Xing BS (2014). Titanium dioxide nanoparticles as carrier facilitate bioaccumulation of phenanthrene in marine bivalve, ark shell (*Scapharca subcrenata*). Environ. Pollut..

[65] Johnston BD (2010). Bioavailability of nanoscale metal oxides TiO_2_, CeO_2_, and ZnO to fish. Environ. Sci. Technol..

[66] Hopper HL (2011). Comparative chronic toxicity of nanoparticulate and ionic zinc to the earthworm *Eisenia veneta* in a soil matrix. Environ. Int..

[67] Gaiser BK (2012). Interspecies comparisons on the uptake and toxicity of silver and cerium dioxide nanoparticles. Environ. Toxicol. Chem..

[68] Bemrah N, Sirot V, Leblanc JC, Volatier J (2009). L.Fish and seafood consumption and omega 3 intake in French coastal populations: CALIPSO survey. Public Health Nutr..

[69] U.S. Department of Health and Human Services and U.S. Department of Agriculture. 2015–2020 Dietary Guidelines for Americans, http://health.gov/dietaryguidelines/2015/guidelines/ Assessed: 2, 2017.

[70] World Health Organization. Safety of Pyrethroids for Public Health Use, http://apps.who.int/iris/bitstream/10665/69008/1/WHO_CDS_WHOPES_GCDPP_2005.10.pdf/ Assessed: 2, 2017.

[71] McDonald, J. H. *Handbook of Biological Statistics* (Sparky House Publishing, Maryland, 2014).

