# Supplementary material for: Ocean acidification increases the accumulation of titanium dioxide nanoparticles (nTiO2) in edible bivalve mollusks and poses a potential threat to seafood safety
Source: Sci Rep. 2019 Mar 5;9:3516. doi: 10.1038/s41598-019-40047-1 (PMC6401146; doi:10.1038/s41598-019-40047-1)
Supplement: Supplementary file 1 — Supporting information [file 41598_2019_40047_MOESM1_ESM.doc]

**Ocean acidification increases the accumulation of titanium dioxide nanoparticles (nTiO_2_) in edible bivalve mollusks and poses a potential threat to seafood safety**

Wei Shi^1^; Yu Han^1^; Cheng Guo^1^; Wenhao Su^1^; Xinguo Zhao^1^; Shanjie Zha^1^; Yichen Wang^1^; Guangxu Liu^1,*^

^1^College of Animal Sciences, Zhejiang University, Hangzhou, P.R. China

**
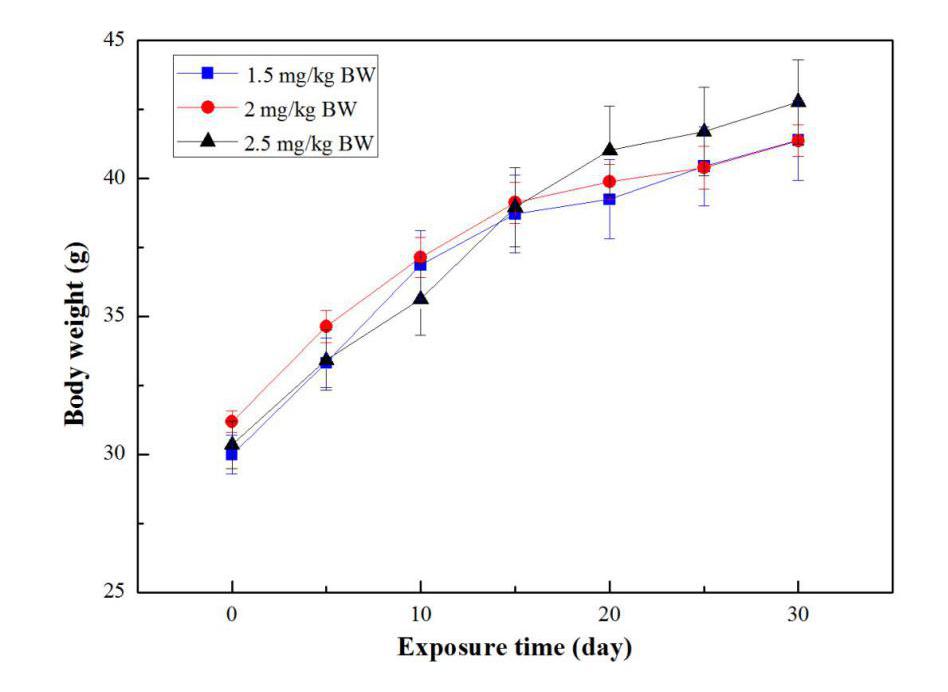
**

**Figure S1. Body weights of mice after oral exposure to nTiO_2_ for 30 days.** * indicate significant difference from group pH 8.1.

**
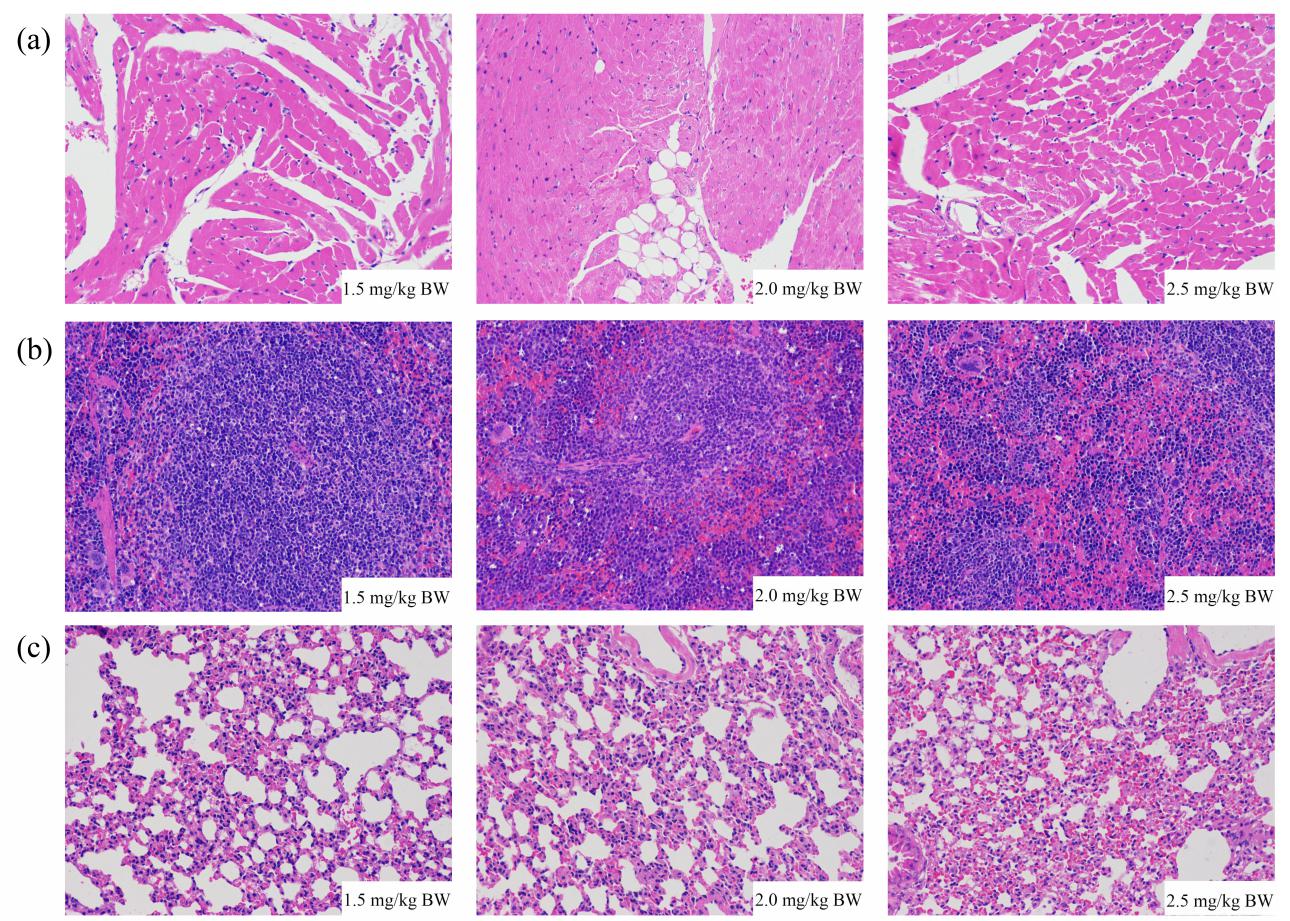
**

**Figure S2. Representative histological photomicrographs of heart (a), spleen (b), and lung (c) in mice after exposure to nTiO_2_ at different doses corresponding to daily intake of nTiO_2_-contaminated seafood at different *p*CO_2_ levels for 30 days (HE × 200).** Three mice in each group were used for histological examination (n=3).


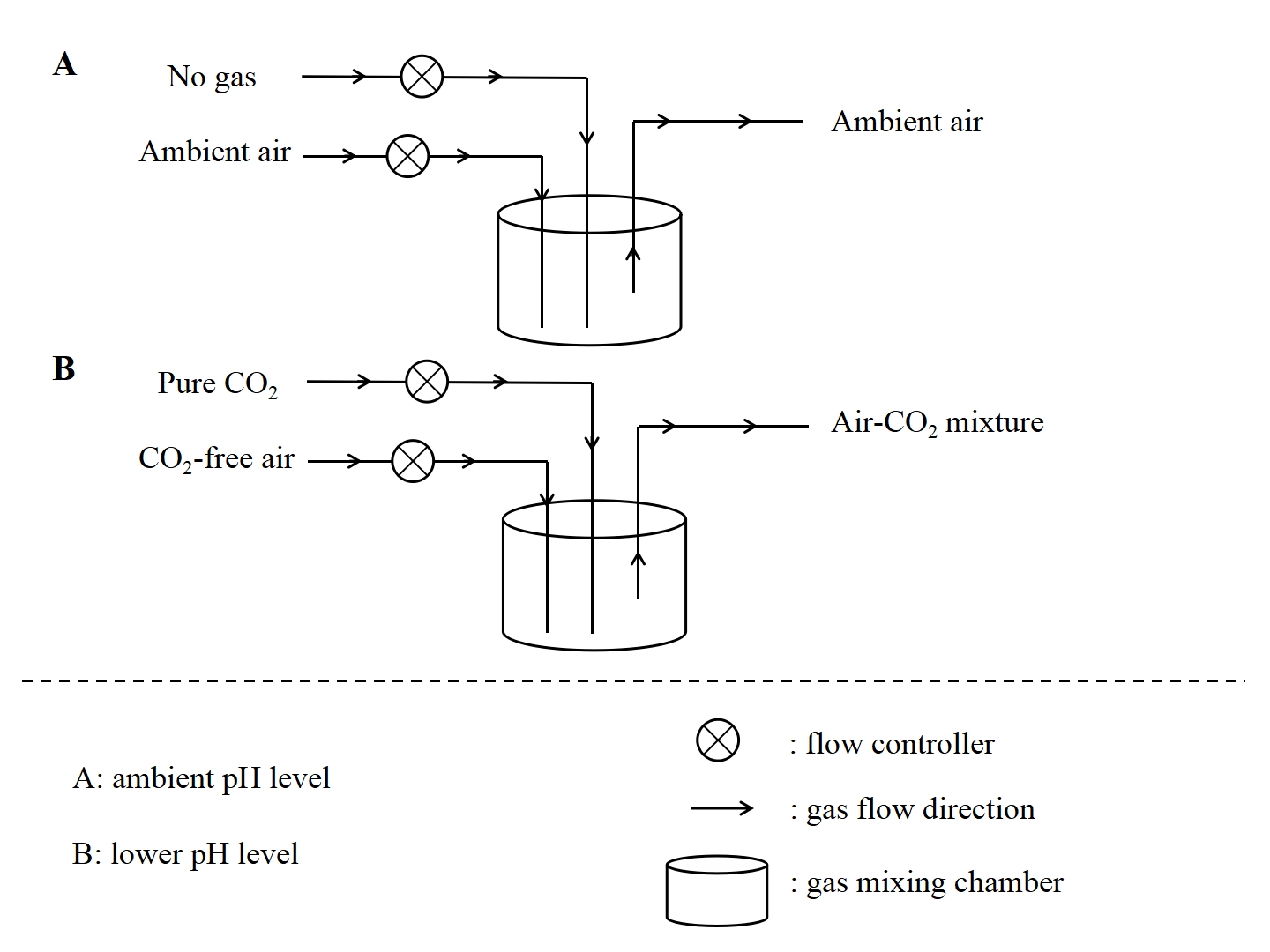


**Figure S3. Schematic diagram of flow controllers and gas mixing setup.**

**
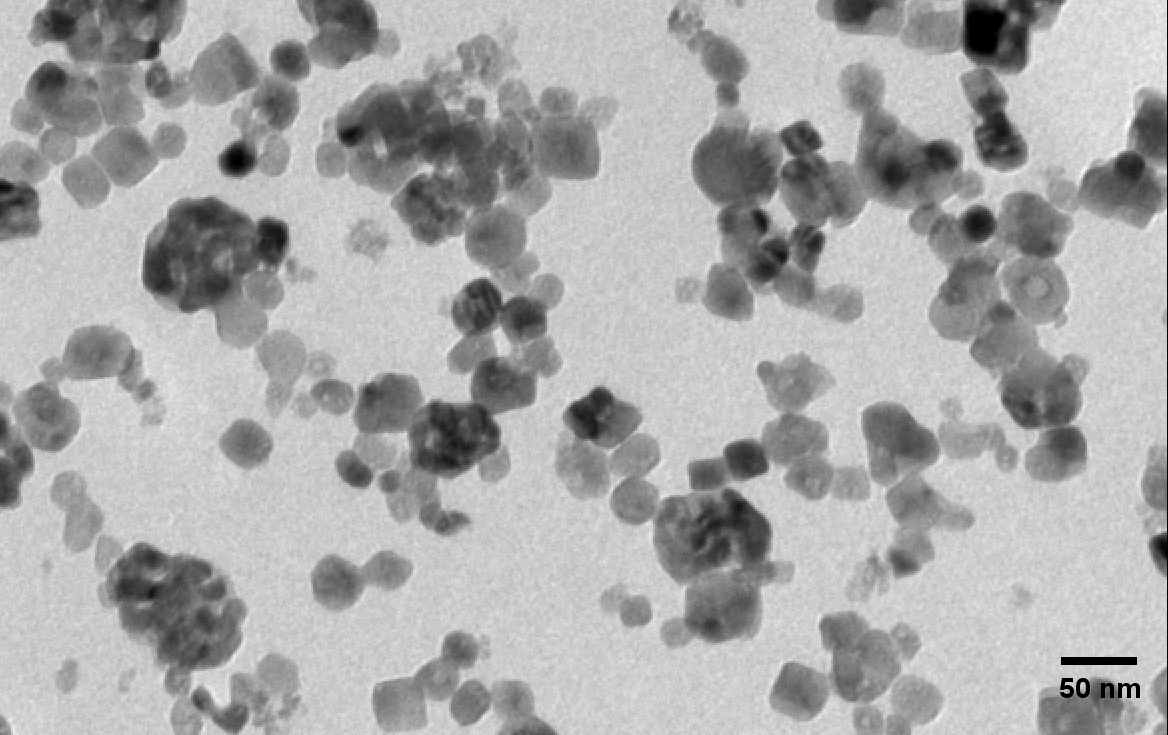
**

**Figure S4. TEM micrograph of titanium dioxide nanoparticles.**

**Table S1. Summary of the impacts of pHs on accumulation of nTiO_2_ in different tissues of three bivalve species using mixed effect linear model.**

| Species | ***M. meretrix*** | | | ***C. sinensis*** | | | ***T. granosa*** | | |
| --- | --- | --- | --- | --- | --- | --- | --- | --- | --- |
| Factors | Gill | Foot | Mantle | Gill | Foot | Mantle | Gill | Foot | Mantle |
| PH | F_2,44_=644.46  *P*<0.01 | F_2,44_=199.07  *P*<0.01 | F_2,44_=188.23  *P*<0.01 | F_2,44_=439.35  *P*<0.01 | F_2,44_=500.70  *P*<0.01 | F_2,44_=250.97  *P*<0.01 | F_2,44_=232.36  *P*<0.01 | F_2,44_=259.51  *P*<0.01 | F_2,44_=974.29  *P*<0.01 |
| Tanks | F_2,8_=0.24  *P*=0.65 | F_2,8_=0.01  *P*=0.97 | F_2,8_=0.03  *P*=0.88 | F_2,8_=0.01  *P*=0.92 | F_2,8_=0.05  *P*=0.83 | F_2,8_=0.14  *P*=0.73 | F_2,8_=0.05  *P*=0.84 | F_2,8_=0.01  *P*=0.92 | F_2,8_=1.41  *P*=0.29 |

**Table S2.** **Organ coefficients of mice after oral exposure to nTiO_2_ for 30 days (mean ± SE).** BW: body weigh; * indicate significant difference from group pH 8.1.

| Group | Exposure dose | BW (g) | Heart/BW (mg/g) | Liver/BW (mg/g) | Spleen/BW (mg/g) | Lung/BW (mg/g) | Kidney/BW (mg/g) |
| --- | --- | --- | --- | --- | --- | --- | --- |
| pH 8.1 | 1.5 mg/kg BW | 41.38 ± 1.45 | 5.14 ± 0.37 | 38.86 ± 2.34 | 3.04 ± 3.04 | 5.25 ± 0.27 | 13.68 ± 0.70 |
| pH 7.8 | 2 mg/kg BW | 44.39 ± 2.12 | 6.04 ± 0.55 | 43.71 ± 1.49 | 4.04 ± 0.56 | 5.16 ± 0.41 | 15.18 ± 0.98 |
| pH 7.4 | 2.5 mg/kg BW | 42.77 ± 1.53 | 5.16 ± 0.44 | 39.53 ± 1.69 | 4.47 ± 0.87 | 5.18 ± 0.17 | 15.71 ± 1.38 |
